# Thoracic Packing Failure due to Occult Diaphragmatic Injury after Empyema Surgery

**DOI:** 10.70352/scrj.cr.26-0173

**Published:** 2026-07-01

**Authors:** Makoto Tatsumi, Tomonari Oki, Shuhei Iizuka, Toru Nakamura

**Affiliations:** Department of General Thoracic Surgery, Seirei Hamamatsu General Hospital, Hamamatsu, Shizuoka, Japan

**Keywords:** intrathoracic gauze packing, damage control surgery, diaphragm, empyema, thoracic surgery, video-assisted, intrathoracic hemorrhage

## Abstract

**INTRODUCTION:**

Intrathoracic gauze packing is an established damage control technique for life-threatening thoracic hemorrhage in trauma settings. However, its role in nontraumatic coagulopathic bleeding remains unclear, and the mechanisms underlying packing failure have not been well characterized. We report a case in which intrathoracic packing failed due to an unrecognized diaphragmatic injury, highlighting an important limitation of this strategy.

**CASE PRESENTATION:**

An 82-year-old man with chronic kidney disease, diabetes mellitus, and hypoalbuminemia underwent emergency video-assisted thoracoscopic decortication for organized empyema. During surgery, he developed severe intraoperative coagulopathic bleeding in the setting of sepsis. Although the thoracic cavity was temporarily closed, persistent hemorrhage necessitated re-exploration, and intrathoracic gauze packing was undertaken as a damage control strategy. Postoperative imaging suggested adequate filling of the pleural cavity; however, hemodynamic instability persisted. Exploratory laparotomy revealed a previously unrecognized laceration of the left diaphragm, through which the intrathoracic packing gauze had prolapsed into the abdominal cavity. The diaphragmatic injury was repaired, hemostasis was achieved, and the patient subsequently recovered with intensive care support.

**CONCLUSIONS:**

Intrathoracic gauze packing may serve as a valuable temporizing measure for severe coagulopathic intrathoracic bleeding, even in nontraumatic settings, provided that diaphragmatic integrity is preserved. When adequate tamponade cannot be achieved, an occult diaphragmatic injury should be actively suspected. Because such injuries are often difficult to diagnose radiologically in the absence of herniation, early surgical exploration may be warranted. Awareness of this potential mechanism of packing failure may facilitate timely intervention and improve outcomes in critically unstable patients.

## Abbreviations

CKD
: chronic kidney disease
DCS
: damage control surgery
DIC
: disseminated intravascular coagulation

## INTRODUCTION

Empyema is a serious pleural infection that often requires surgical intervention when antimicrobial therapy and chest tube drainage fail to achieve infection control and lung re-expansion.^1–3)^ Patients with empyema frequently have significant comorbidities such as CKD, hypoalbuminemia, and diabetes mellitus, which are associated with increased postoperative morbidity and mortality, particularly in older individuals.^4–7)^ Sepsis frequently complicates empyema, and dysregulated interactions between systemic inflammation and coagulation may lead to the consumption of coagulation factors and platelets, ultimately resulting in DIC.^8,9)^ CKD is an independent risk factor for postoperative bleeding.^10–12)^ Progressive septic DIC is characterized by diffuse oozing from dissected pleural surfaces, rendering conventional surgical hemostasis challenging.^13,14)^ Gauze packing as part of DCS is an established strategy for managing severe hemorrhage associated with physiologic derangement.^15–17)^ This strategy, initially developed in trauma surgery, has subsequently been extended to selected nontraumatic abdominal conditions.^18–21)^ However, its application to nontraumatic thoracic surgery, particularly for coagulopathic intrathoracic bleeding after empyema surgery, has been rarely reported. Effective intrathoracic gauze packing relies on adequate counter-pressure within the thoracic cavity, in which an intact diaphragm plays a crucial role in maintaining the tamponade effect. In this setting, diaphragmatic injury represents a critical but easily overlooked complication, particularly in the presence of coagulopathic bleeding. Such injuries are notoriously difficult to diagnose unless accompanied by an overt hernia. Even small defects may become clinically significant in a delayed fashion in the presence of increased intrathoracic pressure, such as during positive-pressure ventilation, potentially resulting in serious complications.^22–25)^ In this report, we describe a case of severe postoperative coagulopathic bleeding after empyema surgery requiring intrathoracic gauze packing. Because the expected hemostatic effect was not achieved, further exploration revealed an occult diaphragmatic injury.

## CASE PRESENTATION

An 82-year-old man with CKD, diabetes mellitus, hypertension, dyslipidemia, and hypoalbuminemia presented with fever and cough after approximately 1 month of anorexia and generalized fatigue. Chest CT demonstrated a multiloculated left pleural effusion with marked pleural thickening, findings consistent with organized empyema (**Fig. 1A** and **1B**). Elective surgery was initially planned. However, acute deterioration of renal function, reflected by an increase in serum creatinine from 0.93 mg/dL on admission to 4.72 mg/dL on hospital day 6, necessitated urgent surgical intervention. Video-assisted thoracoscopic decortication was performed^26)^ (**Fig. 2A**). A 5-cm access incision and a camera port were placed in the 6th and 8th intercostal spaces, respectively, at the highest points of the thoracic cavity. Dense fibrous adhesions were noted between the lung and the parietal pleura, particularly around the diaphragm (**Fig. 2B**). During adhesiolysis and drainage of the abscess of cavity, the patient’s hemodynamic and respiratory status rapidly deteriorated, consistent with septic shock. Four hours after the start of surgery, he remained hemodynamically unstable despite vasopressor support with noradrenaline at 0.7 µg/kg/min (γ), adrenaline at 0.3γ, and vasopressin at 3 units/h. Blood pressure was difficult to maintain, reaching 69/48 mmHg, with a persistent tachycardia of 144/min. Preoperative laboratory tests showed a platelet count of 416 × 10^3^/µL and a prothrombin time and international normalized ratio (PT-INR) of 1.20, which deteriorated intraoperatively to 314 × 10^3^/µL and 1.41, respectively, suggesting an evolving coagulopathy with concern for impending DIC. Further supporting the diagnosis of sepsis-induced coagulopathy, additional laboratory evaluation revealed an elevated D-dimer level of 5.8 µg/mL. The patient’s Sequential Organ Failure Assessment score was 18, and *Prevotella nigrescens* was subsequently isolated from both blood and purulent fluid cultures. Although the cumulative blood loss was 900 mL, persistent diffuse oozing from the dissected pleural surfaces raised concern for progressive coagulopathy associated with septic DIC. Given the inability to achieve definitive hemostasis, we decided to abandon complete decortication. Following irrigation with 15000 mL of saline and empiric intrathoracic disinfection with gentian violet,^27,28)^ the procedure was concluded with 2 chest drains placed. The patient was transferred to the ICU intubated and requiring high-dose vasopressor support. Despite massive transfusion, his cardiorespiratory status remained unstable, with persistent hemorrhagic output from the chest drains. Within 1 h of ICU admission, the cumulative chest tube output exceeded 2000 mL. This was accompanied by worsening metabolic acidosis (pH 7.16, lactate 141 mg/dL) and marked coagulopathy (platelet count 4.4 × 10^4^/μL and PT-INR 1.50), resulting in hemorrhagic shock and prompting an emergent thoracotomy at the bedside. During re-exploration, severe coagulopathy prevented the identification of a discrete bleeding source and precluded definitive surgical hemostasis. We proceeded with DCS using intrathoracic gauze packing. A total of 11 Mikulicz gauze packs (30 × 60 cm) were placed throughout the left hemithorax—including the apex, paravertebral region, anterior and lateral lung surfaces, and around the diaphragm—to achieve effective counterpressure, followed by temporary chest closure. Although postoperative imaging suggested adequate filling of the pleural cavity (**Fig. 3**) with no active bleeding from the chest drains, high-dose vasopressors and continued transfusion remained necessary. Four hours after re-exploration, the patient developed marked abdominal distension accompanied by refractory metabolic acidosis and worsening hyperlactatemia, with an arterial pH of 6.99 and a lactate level of 186 mg/dL. Nonocclusive mesenteric ischemia was suspected to be precipitated by high-dose vasopressor support, prompting an emergent exploratory laparotomy. Intraoperative findings revealed bloody ascites and violet discoloration of the bowel and mesentery, attributable to prior irrigation with gentian violet during thoracic lavage, with no frank evidence of irreversible intestinal ischemia (**Fig. 4A**). Further exploration revealed a laceration of the left hemidiaphragm, through which the intrathoracic packing gauze had prolapsed into the abdominal cavity (**Fig. 4B**). The diaphragm injury was repaired with interrupted sutures, leading to immediate hemodynamic improvement. Thirty-two hours after the exploratory laparotomy (44 h after the initial surgery), planned thoracic re-exploration was performed after confirmation of improved coagulation parameters (**Fig. 5**). Removal of the packing gauze revealed pulsatile bleeding from the cut surface of the thickened pleural peel on the diaphragm, which was successfully controlled by cauterization. With no additional bleeding sources identified, definitive chest closure was performed. Vasopressors were discontinued on the day of definitive closure, and no further transfusions were required. Subsequent CT imaging (POD 5 and POD 22 after definitive closure) and clinical follow-up confirmed the absence of recurrent intrathoracic bleeding or empyema, and all chest drains were removed on POD 29 after definitive closure. However, the left empyema ultimately recurred subsequently, followed by progressive multiple organ failure, and the patient died on POD 42 after definitive closure.

**Fig. 1:**
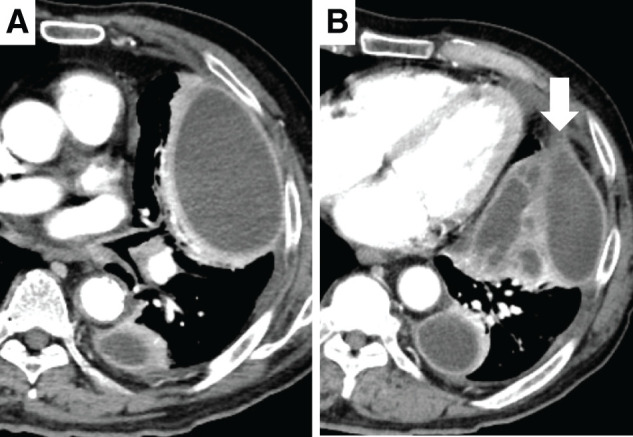
Contrast-enhanced CT findings. (**A**) Axial contrast-enhanced CT demonstrates multiple loculated fluid collections with diffuse pleural thickening in the left hemithorax. (**B**) Marked inflammatory thickening is evident particularly along the diaphragm (arrow).

**Fig. 2:**
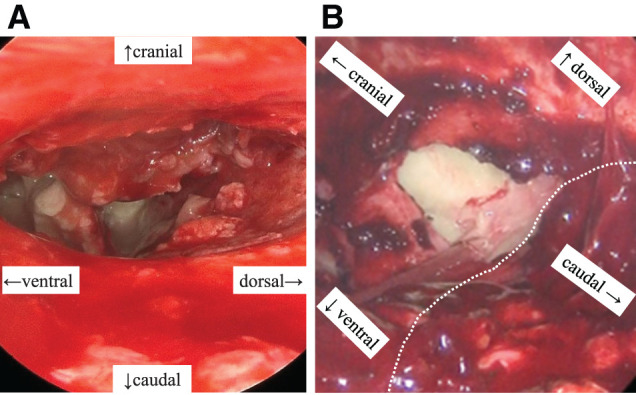
Intraoperative findings during video-assisted thoracoscopic decortication. (**A**) Thoracoscope findings revealed marked pleural thickening and dense adhesions narrowing the thoracic cavity. (**B**) All abscess cavities were opened and drained; adhesions were particularly dense around the diaphragm (dotted line). Extensive inflammation rendered normal anatomy and tissue planes indistinct.

**Fig. 3:**
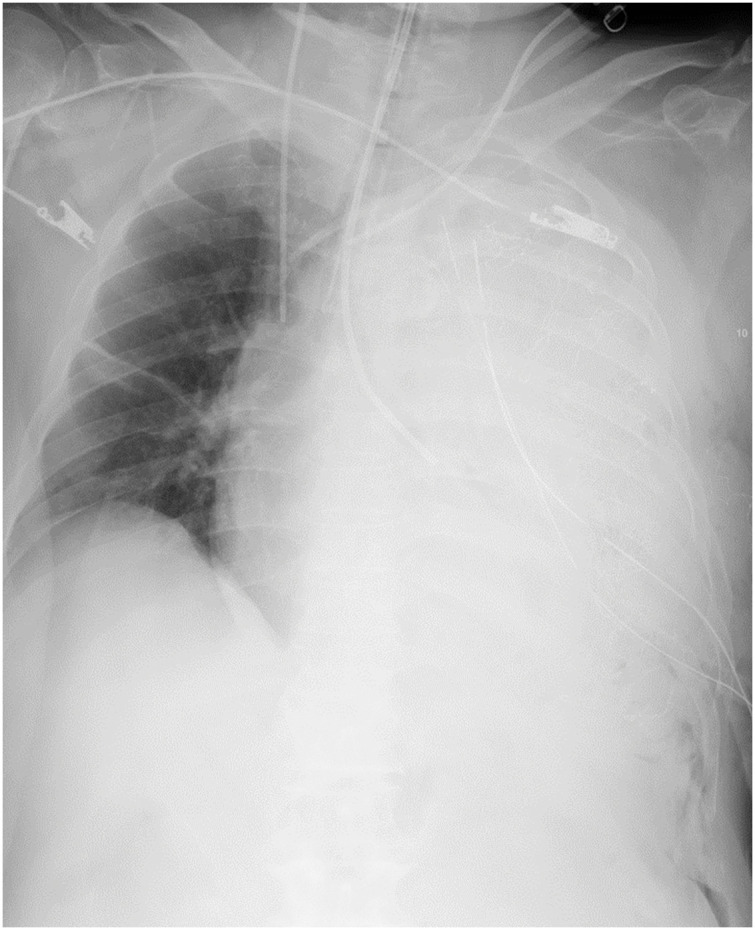
Chest radiograph obtained immediately after intrathoracic gauze packing shows extensive radiopaque packing material occupying the left hemithorax, indicating sufficient filling of the pleural space.

**Fig. 4:**
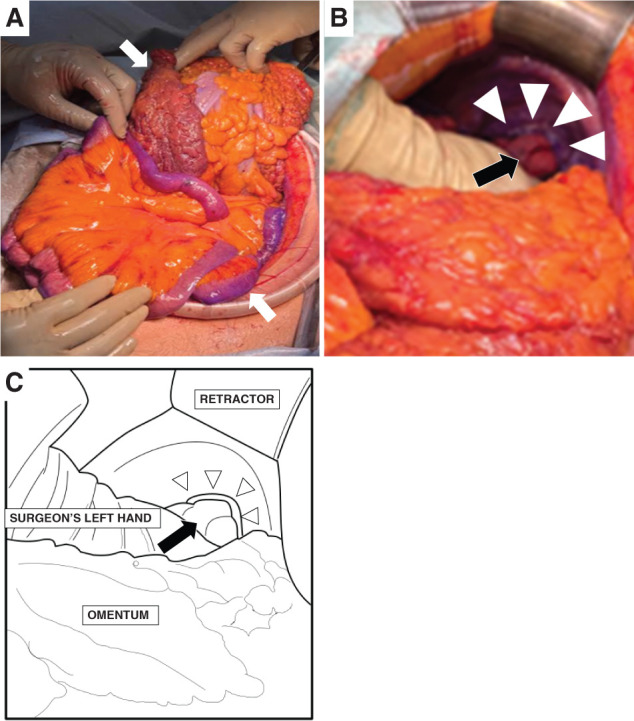
Intraoperative findings at exploratory laparotomy. (**A**) The small intestine, mesentery, and greater omentum are stained with gentian violet (arrows). (**B**) A laceration of the left diaphragmatic is identified (arrowheads), through which the Mikulicz gauze has prolapsed into the abdominal cavity (arrow). (**C**) A schematic illustration of (**B**) shows a diaphragmatic laceration (arrowheads) and the prolapsed gauze (arrow).

**Fig. 5:**
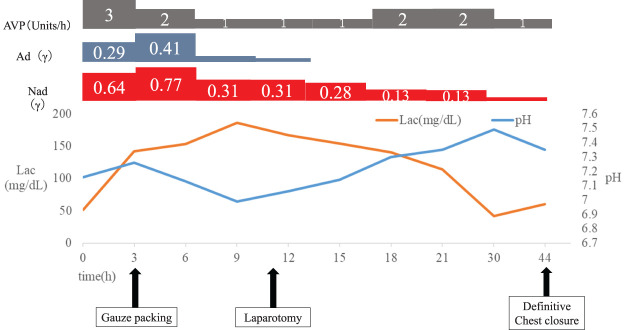
Time course of vasopressor requirements and changes in arterial pH and lactate levels from the initial surgery to definitive chest closure. Catecholamine doses are expressed in µg/kg/min and vasopressin as Units/h. Time zero (0 h) represents ICU admission after the initial surgery. Arterial blood gas analyses were not performed between 21 and 30 h. Ad, adrenaline; AVP, vasopressin; Lac, lactate; Nad, noradrenaline

## DISCUSSION

This case illustrates how intrathoracic packing can fail in the presence of an unrecognized diaphragmatic injury. In this patient, empyema progressed to severe sepsis and coagulopathy, ultimately requiring DCS, during which an unrecognized diaphragmatic laceration was present. In this coagulopathic state, tissues are inherently prone to bleeding, and negative pressure by the chest tube, as well as surgical intervention, may further increase the risk of bleeding. In the present case, these factors were compounded by septic shock, sepsis-induced DIC, and the acute deterioration of preexisting CKD. Although intrathoracic gauze packing is an established DCS technique in severe thoracic trauma,^15–17)^ it has rarely been applied to nontraumatic settings. The hemostatic effect of intrathoracic gauze packing depends on the stable counterpressure within a closed thoracic compartment bounded by relatively noncompliant structures, where the applied pressure exceeds local intravascular and tissue pressures.^29)^ However, even a small diaphragmatic injury can disrupt this pressure equilibrium and allow blood and packing material to prolapse into the abdominal cavity, as shown in our case. Surgical decortication for organized empyema involves extensive adhesiolysis in the setting of dense inflammatory adhesions and distorted anatomy, which may obscure the normal tissue planes and predispose the patient to inadvertent diaphragmatic injury. Decortication using a sharp curette in the setting of coagulopathy may have caused inadvertent diaphragmatic injury. In the present case, the diaphragmatic injury was overlooked during the initial surgery because of the ongoing coagulopathic bleeding. Postoperative positive-pressure ventilation and sustained mechanical stress from intrathoracic gauze packing may have enlarged the defect, ultimately leading to delayed clinical presentation. This case suggests that the tamponade failure of intrathoracic packing should prompt active suspicion of an occult diaphragmatic injury. Unexplained postoperative abdominal distension following thoracic surgery, particularly in the setting of positive-pressure ventilation, also warrants consideration of this possibility.

Diaphragmatic lacerations are difficult to diagnose radiologically unless accompanied by herniation. Retrospective studies of traumatic diaphragmatic injury have shown that nearly half of cases were not identified on preoperative imaging, underscoring the diagnostic limitations of CT in this setting.^22–25)^ These findings suggest that, when diaphragmatic injury is suspected, prompt surgical exploration is essential for definitive diagnosis and management. The present case underscores that intrathoracic gauze packing can serve as a temporizing strategy for severe bleeding, even in nontraumatic settings, provided that the thoracic compartment remains intact. Tamponade failure should prompt suspicion of occult diaphragmatic injury and early surgical intervention.

## Limitations

Several limitations of this report warrant acknowledgment. First, this is a single case report, and the generalizability of our findings is inherently limited. Second, although intrathoracic gauze packing was employed as a damage control strategy in this case, it is important to recognize that successful tamponade within the thoracic cavity may itself carry inherent physiologic risks. As noted in the literature, bulky intrathoracic packing can elevate intrathoracic pressure and mechanically compress the right heart, hilar pulmonary vessels, and the superior vena cava, potentially resulting in cardiopulmonary compromise.^29)^ Similarly, excessive pressure from thoracic packing has been associated with a risk of thoracic compartment syndrome, analogous to abdominal compartment syndrome in damage control laparotomy.^18)^ In the present case, because the tamponade effect was not achieved due to the diaphragmatic injury, these complications did not occur following the repair and repeat packing. Nevertheless, clinicians should remain vigilant for signs of increased intrathoracic pressure and cardiac compression when employing this technique, and the threshold for reoperation should be low if hemodynamic deterioration persists despite packing.

## CONCLUSIONS

Intrathoracic gauze packing may serve as a temporizing strategy for severe intrathoracic hemorrhage, even in a nontraumatic setting. Preservation of diaphragmatic integrity is a key determinant of effective hemorrhage control within the thoracic cavity. In the present case, the tamponade effect was compromised by a diaphragmatic injury; however, prompt repair of the defect led to rapid hemodynamic improvement, underscoring its critical role. Failure to achieve adequate tamponade should raise suspicion of an occult diaphragmatic injury and warrants early surgical exploration, which may improve outcomes in critically unstable patients.
